# Biomechanical comparison of pin and freeride ski mountaineering bindings in recreational skiers

**DOI:** 10.3389/fspor.2025.1679637

**Published:** 2026-01-12

**Authors:** Isaac Burgess, Samantha Samuels, Tyler Whittier, John Seifert, James Becker

**Affiliations:** Department of Food Systems, Nutrition and Kinesiology, Montana State University, Bozeman, MT, United States

**Keywords:** biomechanics, equipment weight, ski bindings, ski mountaineering, skimo

## Abstract

**Introduction:**

Although increased equipment weight is known to increase the metabolic cost of uphill skiing at a constant grade, the effects of different binding types on ski mountaineer kinematics across varying grades remains unknown. This study compared lower-body kinematics in recreational skiers between those using pin bindings and those using freeride bindings.

**Methods:**

Sixteen participants skied on a treadmill using either pin or freeride bindings mounted to matching skis during 3-min stages at grades of 8% and 15%. Kinematic data were recorded during the final 30 s of each stage. Sagittal-plane joint angles of the lower limbs and torso, along with cycle metrics, were compared across conditions using statistical parametric mapping and linear mixed-effects models.

**Results:**

Compared with pin bindings, freeride bindings showed greater ankle plantarflexion (*p* = 0.05), knee flexion (*p* = 0.02), and hip flexion (*p* = 0.05) near toe-off, although the magnitudes of these differences were small. Freeride bindings also resulted in slower cycle rates (*p* < 0.001, d = 0.34), longer cycle lengths (*p* < 0.001, d = 0.36) and cycle times (*p* < 0.001, d = 0.36), and higher step heights (*p* < 0.001, d = 0.32); however, all differences were small in magnitude and effect size.

**Discussion:**

These findings suggest that while biomechanical differences between binding types are statistically significant, their small differences and effect sizes are unlikely to have practical relevance for uphill travel performance in recreational skiers under laboratory settings.

## 1 Introduction

Ski mountaineering (skimo) is one of the fastest-growing winter sports in the United States, with participation projected to increase nearly threefold from 2019 to 2023, reaching approximately 2.2 million people (1). The increase in popularity has also led to a rise in equipment sales, with skimo-specific gear estimated to become an 11-billion-dollar market by 2032 (2), making it the fastest-growing segment of the skiing industry. Skimo attracts both recreational and competitive participants (3). Recreational participants partake in skimo to escape long lift lines at resorts, exercise in the mountains through the winter, and explore new backcountry terrain (4). From a competitive perspective, skimo continues to gain popularity and is set to make its Olympic debut at the 2026 Milano-Cortina Winter Olympics (3).

**Figure 1:**
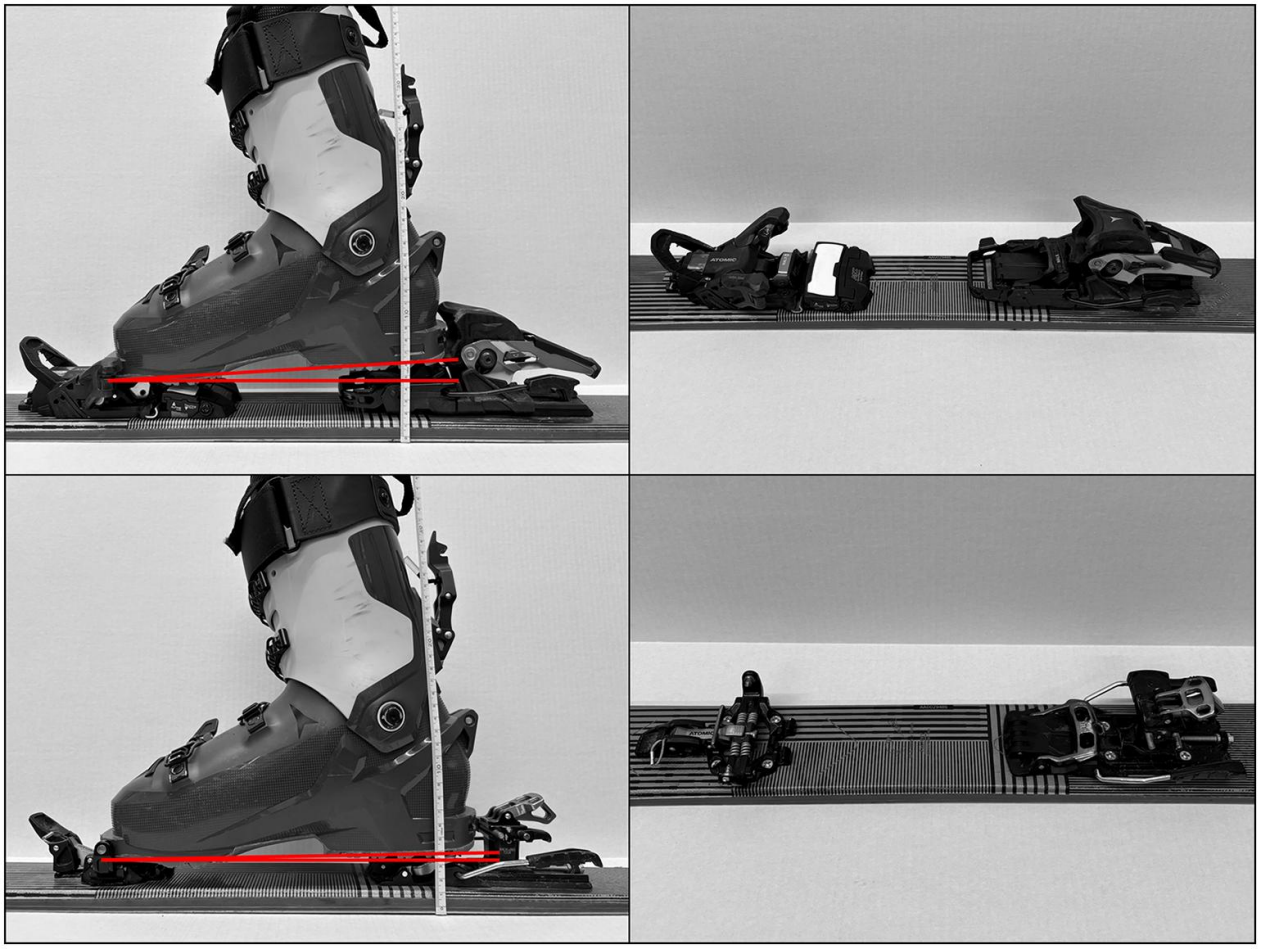
Matching skis mounted with freeride (top row) and pin (bottom row) are shown in walk mode with a boot (left column) and without boot (right column). A ruler displays the greater stack height of the rear piece of the freeride binding compared to the pin binding. The greater delta-angle of the freeride binding is also shown.

Given the increase in skimo popularity, there has been a rapid growth in scientific research investigating factors that influence skimo performance. Several studies have shown that increased binding or boot weight significantly increases the energetic cost (5–8). As a result, almost all skimo athletes use ultralightweight binding designs (3) featuring toe pins that clamp into specialized inserts in the boot toe (9) (Figure 1). This design allows the specialized skimo boot to hinge freely within the binding, allowing for unrestricted ankle rotation and promoting a natural walking-like movement during uphill skinning. For downhill travel, the boot is locked rigidly, and the heel locks into two pins protruding from the binding heel piece, securing the boot to the ski during descent. Despite their lightweight design, several studies have shown that pin (P) bindings do not meet the International Organization for Standardization standards for retention–release values for either alpine skiing or skimo bindings (10, 11), often requiring greater torsional forces to trigger release from the binding (9, 12).

The increased torsional release forces required by pin bindings raise concerns regarding injury while skiing in these bindings (9–12), especially for skiers who prioritize downhill skiing performance. Consequently, many recreational skiers want bindings that permit uphill travel while incorporating the safety features required for typical alpine bindings. In response, manufacturers have developed hybrid freeride (FR) bindings that fully enclose both the toe and heel of the boot during downhill skiing, similar to traditional alpine bindings, but transition to toe pin inserts and a releasable heel for uphill travel (Figure 1). However, freeride bindings are heavier than pin bindings and feature a different design, which may alter skier kinematics during uphill travel.

Previous literature has examined the influence of equipment weight in recreational skimo, finding a positive linear relationship between ankle loading and energetic cost during uphill skinning at a constant grade and speed (8). In addition, two studies comparing different boot and binding systems (6, 7) reported significant increases in vertical mechanical work and energy cost with heavier skimo configurations. However, these studies have two limitations. First, they evaluated older frame-style bindings in which the entire binding system remains attached to the boot during uphill skiing. This aspect of equipment is a key technological upgrade brought on by newer hybrid freeride bindings that only require movement of the boot. Second, while these studies provide some insight into the effect of equipment weight on energetic cost, they did not assess skier biomechanics across conditions, which would likely accompany or drive changes in energy cost.

To date, few studies have investigated the biomechanics involved in skimo, with most focusing on gross cycle kinematic parameters such as stride rate and length (5, 13), and only one study investigating the effects of binding riser height on whole-body kinematics and kinetics (14). Bortolan et al. (5) reported no changes in cycle kinematics with the addition of weight to the ski boot in highly trained ski mountaineers. However, the weight on the boot may not be the same as the weight on the ski, as several studies on walking and running have shown that more distal weight placement results in greater impacts on energetic cost and biomechanics (15–17). In these studies, the addition of mass to the foot, compared with loading the shank, thigh, or an unloaded control, significantly increased cycle length, decreased cycle rate, prolonged swing time, and elevated the energetic cost of movement (15, 17), while also producing small effects on joint ranges of motion at the hip, knee, and ankle (15). Thus, the additional mass of the freeride binding attached at the toe of the boot may cause similar biomechanical changes compared to a pin binding.

An additional factor that may influence skinning biomechanics in pin versus freeride bindings is the difference in stack height, defined as the distance from the ski to the ski boot sole (18). As can be seen in Figure 1, freeride bindings have a higher rear binding stack height than pin bindings, which increases the boot delta angle of the binding (19). An increased delta angle may lead to similar changes in skier biomechanics that have been observed with an increased heel riser height, including decreased cycle length, increased cycle rate, and smaller ranges of motion at the ankle, knee, and hip joints (14). However, the difference in delta angle between pin and freeride bindings is relatively small compared with that produced by using an actual heel riser and therefore may not be enough to cause any significant kinematic changes.

A final consideration when evaluating biomechanical differences between binding types is the potential interaction effect of grade, as grade has been shown to influence other aspects of skimo (14, 20–21), as well as other forms of locomotion, such as running and walking (22–24). Lasshofer et al. (14) found lower-body joint angles, step length, and energy cost were all affected by both riser height and gradient. With increasing grade, cycle length, cycle time, swing time, and stance phase all increased, while cycle rate decreased (14). Also, lower-body joint kinematics varied with grade, with ranges of motion at the ankle, knee, and hip increasing with increasing grade (14). In addition, Praz et al. (20, 21) reported that cycle metrics, energy cost, and efficiency in skimo athletes vary significantly with changes in speed and slope angle. The importance of considering slope angles in locomotion research is further supported by previous walking gait studies showing that walking biomechanics change across gradients (23, 24).

Therefore, the purpose of this study was to compare lower-body joint and cycle kinematics during uphill skiing using pin and freeride bindings across two slope gradients and two speeds in a recreational skier population. We hypothesized that (H1) ankle, knee, and hip ranges of motion would differ minimally between FR binding types across grades, and (H2) FR bindings would result in longer and slower cycle kinematics across grades.

## 2 Materials and methods

### 2.1 Participants

An *a priori* power analysis [G*Power, version 3.1 (25)] was performed for a paired, within-subject comparison of binding type and grade. Based on the magnitude of differences reported by Lasshofer et al. (14), we estimated that, using an alpha level of 0.05 for comparisons of bindings and grade, we would have 80% power to detect a 9.0° difference in joint angles and a 6% difference in cycle kinematics with at least seven subjects. Anticipating smaller effect sizes for binding comparisons than those reported by Lasshofer et al. (14), we recruited approximately twice the required number of participants.

Fifteen skiers (eight men and seven women; age: 22 ± 2 years; height: 1.77 ± 0.08 m; body mass: 71.5 ± 10.2 kg) participated in this study. Participants were recruited via flyers posted at local ski trailheads, ski shops, and on social media. Inclusion criteria included at least 2 years of recreational skimo experience, no history of lower-extremity injury within the previous year, and a boot sole length compatible with the research skis used in the study (262–288 mm for shorter skis or 295–325 mm for longer skis). Exclusion criteria included no prior experience with skimo or less than 4 days of ski mountaineering during the current/most recent ski season, as well as any lower-extremity injury that required surgery within the past 2 years. All participants provided written informed consent and were informed of their right to leave or opt out of the study at any time. The study protocols were approved by the Montana State University Institutional Review Board under protocol no. 1126.

### 2.2 Experimental design

This was a quasi-randomized, within-subject, repeated-measures experimental study. All testing was completed during a single laboratory visit. Prior to testing, participant height and total body mass, measured while wearing all research equipment, clothing, and ski equipment (boots, poles, and each set of skis separately; Table 1), were recorded using an integrated beam scale and stadiometer (Health-O-Meter, Continental Scale Corp., Bridgeview, IL, USA). Participants then completed a 5-min warm-up by skiing at self-selected pace on an oversized ski treadmill (belt dimensions: 2.5 m × 3 m, Fitnex Fitness Equipment Inc., Dallas, TX, USA). Consistent with the study by Lasshofer et al. (14) on biomechanical effects of heel risers, our treadmill skiing protocol consisted of 3-min bouts at 8% and 15% grades, performed at a speed of 1.1 m/s for both grades; trials were conducted using matching skis mounted with either Backland Tour pin (P) or Shift 13MN FR bindings (Figure 1). This speed was selected to match the protocol used by Lasshofer et al. (14), which allowed direct biomechanical comparability and ensured that all participants could maintain a steady and reproducible technique across both grades. Both bindings were manufactured by Atomic (Atomic Austria GmbH, Altenmarkt, Austria) and had masses of 398 and 880 g per binding, respectively. The order of binding type and grade was quasi-randomized across participants, such that participants either received the P or FR binding first and started at either the 8% or 15% grade. To minimize the number of ski changes, both stages for a given binding were completed before changing to the other binding type. All trials were performed using participants' own boots and poles, but with laboratory-provided skis mounted with either the P or FR bindings. The different bindings accounted for approximately 1%–2.5% of the total mass of the skier system (Table 1). For each binding type, two ski lengths were used depending on boot sole length of participants (long: 179 cm, Backland 85 UL, short: 165 cm, Backland 86 SL; Atomic Austria GmBH, Altenmarkt, Austria). Torso and lower-body kinematics were recorded during the final 30 s of each stage of the treadmill skiing protocol.

**Table 1:** Total mass of the skier with all equipment (mean ± SD) and the relative contributions of skis, boots, and poles, and only bindings.

Binding	Total mass of the setup (kg)	Relative mass of skis, boots, and poles (% BW)	Relative mass of bindings only (% BW)
Pin (P)	79.3 ± 10.7	10.9 ± 1.8	1.1 ± 0.2
Freeride (FR)	80.3 ± 10.7	12.3 ± 1.8	2.5 ± 0.3

BW, body weight.

### 2.3 Instrumentation

Whole-body kinematics were recorded during the treadmill skiing protocol using a six-camera motion capture system (Motion Analysis Corp, Rohnert Park, CA, USA) sampling at 120 Hz. Forty retroreflective markers were placed on the torso, pelvis, and lower extremities to create an eight-segment biomechanical model in accordance with ISB recommendations (26, 27). Markers used to define anatomical coordinate systems were placed bilaterally on the acromion processes, anterior and posterior superior iliac spines, medial and lateral femoral epicondyles, medial and lateral boot hinge points, and the mid-toe of the boot. Markers placed on the boot were used to represent the foot anatomical coordinate system but did not necessarily represent true anatomical landmarks of the foot. However, these markers closely aligned with the axis of possible ankle movement in the ski boot (14). Rigid clusters consisting of four non-colinear tracking markers were securely attached to the thigh and shank segments using elastic wrap. Additional tracking markers were placed on the xiphoid process of the sternum, the seventh cervical vertebra, the iliac crests, the approximate base of the fifth metatarsals (crest of the lateral boot curve), and the heel, aligned with the toe marker. Markers used to track ski kinematics were placed on the medial and lateral edges of the ski tips, in front of the toe piece, behind the heel piece, on the ski tail, and markers placed on the treadmill corners defined the treadmill belt as a segment.

### 2.4 Data analysis

Marker trajectories were tracked in Cortex software (Motion Analysis Corp, Rohnert Park, CA, USA), gap-filled, and filtered using a zero-lag, low-pass Butterworth filter with a cutoff frequency of 8 Hz. The marker data was exported to Visual3D (C-Motion, Germantown, MD, USA), and a six degree-of-freedom biomechanical model was constructed for the ankle, knee, hip, and torso. Joint angles were calculated using an XYZ Cardan rotation sequence, expressing the orientation of the distal segment relative to the proximal, where X corresponded to flexion/extension, Y represented abduction/adduction, and Z represented axial rotation.

The gait cycle was defined using the marker coordinates of the ski tip marker in the X-direction (primary travel direction). A single gait cycle was defined as the forward-most point achieved by the ski tip marker until the subsequent forward-most point (5, 28). Assuming negligible sliding between the treadmill belt and base of the ski, a two-phase gait cycle was defined according to previous literature on walking gait (28). The stance phase was defined as the period from the forward-most to the most rearward point of the marker, constituting the time during which the ski was placed on the treadmill belt. The swing phase was defined as the period from the most rearward to the forward-most point of the marker, constituting the time during which the participant advanced the ski forward on the treadmill. Joint angles for the ankle, knee, hip, and torso across each gait cycle were exported to MATLAB 2024b (The MathWorks, Natick, MA, USA), where a custom script normalized each to 100% of the gait cycle and computed an ensemble average across 15 gait cycles for each of the six experimental conditions. The processed data were stored for future statistical analysis.

In Visual3D, the range of motion (ROM) of the ankle, knee, hip, and torso was calculated for each gait cycle and condition by calculating the difference between the maximum and minimum joint angles achieved during each cycle. Cycle metrics were computed for each gait cycle across all conditions and included cycle rate (steps/min), cycle time, cycle length, and stance and swing durations expressed as a percentage of total cycle time. Peak step height during the swing phase was also calculated as the maximum perpendicular distance between the toe marker and the treadmill belt.

### 2.5 Statistical analysis

Temporal differences in joint angle curves across the stance phase between bindings and grades were assessed using statistical parametric mapping (SPM 1D) (29, 30) with a 2 × 2 repeated-measures design implemented in MATLAB. Differences in joint ROM and cycle metrics were assessed using linear mixed-effects models (31). Binding type and grade, as well as their interaction, were included in the models as fixed effects, with participant included as a random effect. When a significant fixed effect was found at the *α* = 0.05 level, *post-hoc* pairwise comparisons were performed with a Bonferroni correction, and Cohen's *d* effect sizes were computed to aid interpretation of the results. Effect sizes were interpreted as small (<0.2), moderate (0.4–0.6), and large (>0.8). The statistical analyses were performed twice: once comparing grades at matched speeds and once comparing grades at matched relative intensities. Except from SPM analyses, all statistical analyses were performed using SPSS version 30 (IBM Corp, Armonk, NY, USA).

## 3 Results

### 3.1 Joint angles

Results from the linear mixed-effects model indicated no significant binding-by-grade interactions for joint ROM (all *p*’s *>* 0.36). A main effect of binding was observed for ankle ROM (*F* = 22.1, *p* *<* 0.001, Table 2), with slightly higher ankle ROM when using the FR binding (*p* *<* 0.001, *d* *=* 0.11). No other main effects of binding were observed for joint ROM. However, significant main effects of grade were found for knee (*F* = 516.0, *p* *<* 0.001), hip (*F* = 686.6, *p* *<* 0.001), and torso ROM (*F* = 15.9, *p* *<* 0.001), with ROM at all three joints being greater at the 15% grade (knee *p* < 0.001, *d* = 0.79; hip *p* < 0.001, *d* = 1.3; torso *p* = 0.03, *d* = 0.25).

**Table 2:** Sagittal-plane ROM values for the ankle, knee, hip, and torso (mean ± standard deviation) averaged across 15 gait cycles during the final 30 s of each trial of the treadmill skinning protocol, compared between bindings and grades.

Joint ROM	Pin (P)		Freeride (FR)		Result
	8%	15%	8%	15%	
Ankle (°)	22.6 ± 5.2	22.3 ± 4.1	23.1 ± 4.7	22.9 ± 4.7	A
Knee (°)	45.0 ± 7.1	51.4 ± 7.5	46.0 ± 7.8	51.3 ± 7.1	B
Hip (°)	64.1 ± 4.0	70.8 ± 4.3	65.5 ± 5.0	70.7 ± 5.0	B
Torso (°)	6.0 ± 1.8	6.6 ± 1.9	6.2 ± 2.1	6.6 ± 1.9	B

A and B indicate significant effects of binding and grade, respectively, at the *p* < 0.05 level.

**Figure 2:**
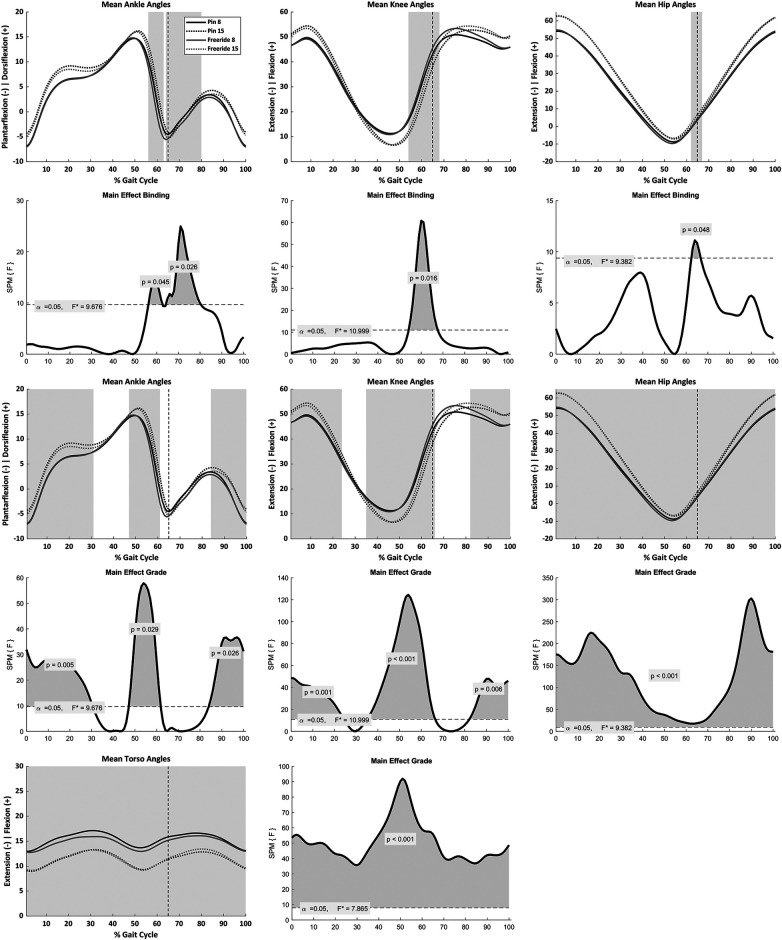
Average joint angles, normalized to 100% of gait cycle, and corresponding SPM{F} plots. The dashed vertical line indicates toe-off. Main effects of binding on ankle, knee, and hip are shown in the first row with corresponding SPM{F} plots displayed in row two. Main effects of grade on ankle, knee, and hip are shown in row three, with corresponding SPM{F} plots in row four. There was no significant effect of binding on the torso at matched speed, however main effect of grade on torso and the corresponding SPM{F} plot are displayed in the fifth row.

The SPM analysis showed no significant binding-by-grade interactions; however, significant main effects of binding and grade were observed. The main effect of binding was primarily isolated to the ankle and knee joints (Figure 2). Compared with the P binding, the FR binding displayed greater plantarflexion during 56%–63% (*p* = 0.05) and 64%–80% (*p* = 0.03) of the gait cycle, corresponding to periods immediately before and after toe-off (Figure 2). Greater knee flexion was observed with the FR binding during 54%–68% of the gait cycle (*p* = 0.02), along with a small cluster indicating reduced hip extension at toe-off (62%–67%, *p* = 0.05) (Figure 2). Significant main effects of grade were observed at the ankle (*F* = 9.676), knee (*F* = 10.999), hip (*F* = 9.382), and torso (*F* = 7.865). At the 15% grade, greater ankle dorsiflexion was observed from initial contact through mid-stance (0%–31% of the gait cycle; *p* < 0.01), immediately before toe-off (47%–62% of the gait cycle; *p* = 0.03), and during mid-to-late swing phase (84%–100% of the gait cycle; *p* = 0.03). Greater knee flexion was observed during 0%–24% (*p* < 0.01) and 82%–100% (*p* < 0.01) of the gait cycle, whereas reduced knee flexion occurred during 35%–67% (*p* < 0.001) of the gait cycle. Finally, at the 15% grade, the hip showed more hip flexion and the torso exhibited reduced flexion across the entire gait cycle (0%–100%, both *p*’s < 0.001).

### 3.2 Cycle metrics

No significant binding-by-grade interactions were observed for any cycle metrics (all *p*’s > 0.20). However, significant fixed effects of binding were found for cycle rate (*F* = 127.8, *p* < 0.001), cycle length (*F* = 130.4, *p* < 0.001), cycle time (*F* = 130.5, *p* < 0.001), percent stance (*F* = 41.8, *p* < 0.001), percent swing (*F* = 163.8, *p* < 0.001), and step height (*F* = 45.2, *p* < 0.001) (Table 3). *Post-hoc* analyses revealed that, compared with the P binding, cycle rate (*p* < 0.001, *d* = 0.34) was lower, cycle length (*p* < 0.001, *d* = 0.36) and cycle time (*p* < 0.001, *d* = 0.36) were longer, percent stance (*p* < 0.001, *d* = 0.21) was shorter, and percent swing (*p* < 0.001, *d* = 0.50) was longer with the FR binding. Step height (*p* < 0.001, *d* = 0.32) was greater in the FR binding compared to the P binding.

**Table 3:** Cycle metrics (mean ± SD) across 15 gait cycles during the final 30 s of each trial of the treadmill skiing protocol, compared between bindings and grades.

Variable	Pin (P)		Freeride (FR)		Result
	8%	15%	8%	15%	
Cycle rate (steps/min)	48.7 ± 2.8	49.2 ± 2.8	47.5 ± 3.3	48.4 ± 2.7	A, B
Cycle length (m)	1.38 ± 0.08	1.37 ± 0.08	1.42 ± 0.10	1.39 ± 0.07	A, B
Cycle time (s)	1.24 ± 0.07	1.22 ± 0.07	1.27 ± 0.08	1.24 ± 0.07	A, B
Percent stance (%)	61.2 ± 4.7	62.3 ± 4.5	60.8 ± 5.2	61.7 ± 4.5	A
Percent swing (%)	38.8 ± 3.0	37.7 ± 2.9	39.2 ± 3.7	38.3 ± 3.0	A, B
Step height (cm)	2.4 ± 1.1	2.6 ± 1.4	2.8 ± 1.1	2.9 ± 1.2	A, B

A and B indicate significant effects of binding and grade, respectively, at the *p* < 0.05 level.

In addition, significant fixed effects of grade were found for cycle rate (*F* = 75.8, *p* < 0.001), cycle length (*F* = 81.0, *p* < 0.001), cycle time (*F* = 81.0, *p* < 0.001), percent swing (*F* = 277.2, *p* < 0.001), and step height (*F* = 6.0, *p* = 0.014) (Table 3). Percent stance (*F* = 0.10, *p* = 0.75) did not display a significant fixed effect of grade. *Post-hoc* analyses revealed that, compared with the 8% grade, cycle rate (*p* < 0.001, *d* = 0.26) was faster, while cycle length (*p* < 0.001, *d* = 0.28), cycle time (*p* < 0.001, *d* = 0.28), and percent swing (*p* < 0.001, *d* = 0.66) were all shorter at the 15% grade. Step height (*p* = 0.014, *d* = 0.12) was also greater at the 15% grade.

## 4 Discussion

The purpose of this study was to compare lower-body joint and cycle kinematics during uphill skiing using P and FR bindings at two slope gradients in a recreational skier population. In partial support of our first hypothesis, ankle ROM was slightly higher when using the FR binding; however, this difference was small and associated with a small effect size. No other joints showed kinematic differences between bindings, and the observed differences between bindings did not vary with grade. Our second hypothesis was also partially supported, as there were longer cycle lengths and slower cycle rates in the FR binding across both grades; however, once again, these differences were relatively small, with small to moderate effect sizes.

### 4.1 Effects of binding

The joint ROM analysis demonstrated that binding type influenced only ankle ROM, with no discernable effects at the knee or hip. This finding shows that the statistically significant waveform differences detected by SPM at the knee and hip did not translate into substantive changes in overall joint excursion and therefore may have limited practical relevance. Importantly, SPM provided information beyond that captured by discrete ROM measures, by revealing that the greater ankle ROM was driven by increased plantarflexion during 56%–63% and 64%–80% of the gait cycle, corresponding to periods immediately before and after toe-off. Although the magnitude of the ankle ROM difference was small, this study highlights the value of combining discrete variables with SPM to identify specific phases of the gait cycle responsible for statistically significant kinematic differences. Future biomechanical analyses of skimo would benefit from adopting this integrated approach, particularly in cases where discrete measures alone may fail to reveal meaningful temporal features of movement patterns (29, 30).

One possible explanation for the increased ankle plantarflexion observed around toe-off is the substantially greater mass of the FR binding compared with the P binding (the Backland Tour pin weighs 389 g, while the Shift 13 MN freeride weighs 880 g, just over twice as much). Because both bindings attach at the same toe pivot point (Figure 1), they share the same lever arm for the ski-binding system relative to the ankle joint. However, the greater mass of the FR binding generates a larger external plantar flexion moment as the ski is lifted during toe-off and early swing. If the ankle dorsiflexor muscles do not fully compensate for this increased external moment, an increase in ankle plantarflexion would be expected. This explanation is consistent with the findings of Browning et al. (15), who demonstrated that moderate increases in foot load significantly increase plantar flexor demand without a corresponding increase in dorsiflexor muscle activity. In addition, the added distal mass lowers the center of mass of the leg and increases the moment of inertia of the shank–foot complex, thus requiring greater plantarflexor torque to initiate swing (15, 32). Prior gait studies have reported similar mechanical effects when mass is added to the foot, including increased plantarflexor demand accompanied by relatively small changes in ankle kinematics (15, 32). Although the magnitude of ankle ROM differences was minimal, this mechanism may help explain the specific time periods of increased ankle plantarflexion identified by the SPM analysis.

The greater mass of the FR binding also provides a possible explanation for the statistically significant changes in cycle metrics. An increased moment of inertia would not only slow the initial acceleration of the limb during swing phase but also make deceleration more difficult once the limb is in motion. Consequently, the limb would travel further before foot contact, resulting in longer cycle lengths and reduced cycle rates. These findings closely align with those from walking and running studies in which mass was added to the foot (15, 16, 32). They also align with previous research in cross-country skiing and running, which shows that small or modest increases in distal loads produce small but detectable changes in timing (17, 33), whereas large distal loads (>4 kg) elicit changes in both cycle characteristics and kinematics (15, 32). In contrast, Bortolan et al. (5) observed that adding mass to the boot of competitive skimo athletes did not meaningfully alter their cycle kinematics, suggesting that higher-level athletes may compensate more effectively for small increases in distal loading. Nonetheless, Bortolan et al. (5) also concluded that the magnitude of these changes was unlikely to influence performance among recreational skiers, a conclusion that aligns with the results of the current study. Thus, although the ∼0.5 kg increase in mass associated with the FR binding was sufficient to generate statistically significant changes in gross cycle kinematics in this recreational cohort, the practical impact of these changes is likely minimal.

In addition to mass, binding geometry may also have contributed to the observed differences. The FR binding has a slightly greater stack height and delta angle compared to the P binding (Figure 1), which could theoretically influence ankle posture and cycle timing in a manner similar to increased heel riser height. However, the only study to date examining heel riser height reported shorter cycle lengths and higher cycle rates with increased heel elevation (14), which is opposite to the findings of the current study. This discrepancy suggests that, in the current study, binding mass exerted a stronger influence on gait mechanics than the relatively small difference in delta angle.

Overall, the increased mass of the FR binding provides the most consistent explanation for the observed changes in both ankle kinematics and cycle metrics. Although these effects were statistically detectable, their small magnitudes, particularly at the joint level, indicate that they are likely of limited practical consequences for most recreational skiers, unless substantially heavier bindings, such as frame bindings, are used. While this study focused on recreational skiers, the minimal kinematic differences, combined with prior evidence that competitive skimo athletes compensate more effectively for equipment modifications (5), suggest that binding-related biomechanical effects would likely be even smaller in elite populations.

### 4.2 Effects of grade

The effects of grade on joint angles observed in the current study are consistent with findings from previous treadmill walking research (23, 24). When considering these findings alongside our results, we see that as grade increases during both walking and uphill treadmill skiing, there is reduced hip extension during stance, greater knee flexion at initial contact, increased ankle dorsiflexion at heel strike, and reduced plantar flexion at toe-off. These similarities likely arise because uphill treadmill skinning does not permit a glide phase and therefore shares key mechanical features with uphill walking gait. Direct comparisons between treadmill skinning and incline walking in future studies may help clarify the extent to which gait adaptations to grade generalize across modalities and identify any skimo-specific strategies that are independent of glide mechanics.

While the extent to which treadmill skinning without glide replicates uphill walking remains to be determined, the effects of grade on cycle metrics observed in the current study are consistent with previous findings from both ski treadmill and on-snow investigations (20, 21). In their treadmill study, Praz et al. (21) reported that as grade increased from 10% to 24% while speed was held constant, elite skimo athletes exhibited shorter cycle lengths, higher cycle rates, and increased stance times. Similar results were observed when the protocol was repeated on snow (20). The findings of the current study mirror this pattern, with steeper grades resulting in shorter cycle lengths and higher cycle rates. These consistent results reflect established mechanical adaptations to increased incline. Taken together, the agreement between treadmill and on-snow studies suggests that the grade-related changes in cycle metrics observed here likely reflect general adaptations to steeper gradients rather than artifacts specific to treadmill skinning.

### 4.3 Limitations

Interpretation of the kinematic differences between bindings observed in this study should be made with caution, as the magnitudes of these changes fall within the typical measurement error of marker-based motion capture systems. Previous studies on walking gait in healthy adults have reported approximately 5° differences as the threshold for minimally detectable changes in sagittal-plane kinematics (34). In contrast, the binding-related differences observed in the current study, whether detected through discrete variables or SPM, were small (i.e., ankle ROM differences of 1° or less and step height differences of ∼0.2 cm). These values are likely below the minimal detectable change of the measurement system and therefore may have limited practical relevance. In addition, although step height is reported to the nearest millimeter, this value represents the average of all participants, and the level of numerical resolution may exceed the practical accuracy of marker-based motion capture. As such, step height values should be interpreted qualitatively rather than as precise measurements.

Also, the mechanical explanations attributing increased ankle joint ROM to external moments should be interpreted with caution, as the absence of ground reaction force measurements prevented our ability to quantify joint moments during treadmill skinning. Although previous studies have shown that adding mass to the foot increases plantar flexor demand and alters muscle activation patterns (15), the present study cannot confirm such kinetic responses without accompanying force or electromyography data. Furthermore, musculoskeletal modeling research suggests that increased distal mass may increase muscle activity in the upper leg, thus contributing to higher metabolic costs of locomotion (16). Whether similar effects occur in skimo with heavier equipment remains unknown and requires future use of synchronized kinematic, kinetic, and electromyography data for a more complete assessment of how equipment mass influences joint kinetics, muscle demands, and overall energetic cost.

From a methodological perspective, an important consideration when interpreting the results of this study is the use of a ski treadmill without climbing skins. Pilot testing revealed that, without skins on the ski base, participants began to slip at grades steeper than ∼20%, which restricted the range of inclines that could be safety tested. Although the grades used in the current study (8% and 15%) represent meaningful differences, they are relatively moderate compared with the steeper slopes often encountered during skimo touring. To address limitations associated with treadmill belt slippage, several authors have incorporated climbing skins or classic roller skis to increase traction and prevent backward sliding (5, 13). In the present study, skins were not used because pilot testing suggested that their presence or lack thereof did not substantially alter the feel of the uphill treadmill skinning. Nonetheless, this equipment configuration may limit the ecological validity of our findings, and future studies should systematically evaluate different treadmill-based skimo setups (i.e., bare ski bases, climbing skins, roller skis) to determine their reliability and comparability to on-snow biomechanics. Such on-snow studies should incorporate steeper grades where feasible and include an assessment of downhill performance, which may be a more critical factor than uphill performance for recreational binding choice.

## 5 Conclusion

The findings of this study indicate that sagittal-plane joint kinematics and cycle metrics differ minimally between P and FR bindings during uphill treadmill skinning. These results are particularly relevant for recreational skiers who often prioritize downhill performance and safety features when selecting bindings. Although the FR bindings are substantially heavier, the additional mass did not produce significant alterations in uphill movement patterns at the tested grades. Combined with the findings of previous studies, the small kinematic differences observed when using a heavier binding are unlikely to influence the uphill technique for recreational skimo athletes ascending at moderate intensities.

## Data Availability

The raw data supporting the conclusions of this article will be made available by the authors, without undue reservation.
